# Grow up, be persistent, and stay focused: keys for solving foraging problems by free-ranging possums

**DOI:** 10.1093/beheco/arad054

**Published:** 2023-06-28

**Authors:** Hannah Harris, Katie K Y Wat, Peter B Banks, Aaron Greenville, Clare McArthur

**Affiliations:** School of Life and Environmental Sciences, The University of Sydney, Heydon-Laurence Building (A08), Sydney, NSW 2006, Australia; School of Life and Environmental Sciences, The University of Sydney, Heydon-Laurence Building (A08), Sydney, NSW 2006, Australia; School of Life and Environmental Sciences, The University of Sydney, Heydon-Laurence Building (A08), Sydney, NSW 2006, Australia; School of Life and Environmental Sciences, The University of Sydney, Heydon-Laurence Building (A08), Sydney, NSW 2006, Australia; School of Life and Environmental Sciences, The University of Sydney, Heydon-Laurence Building (A08), Sydney, NSW 2006, Australia

**Keywords:** animal personality, behavioral flexibility, innovation, learning, urbanization, wildlife

## Abstract

Individuals within a species often vary in both their problem-solving approach and ability, affecting their capacity to access novel food resources. Testing problem-solving in free-ranging individuals is crucial for understanding the fundamental ecological implications of problem-solving capacity. To examine the factors affecting problem-solving in free-ranging animals, we presented three food-extraction tasks of increasing difficulty to urban common brushtail possums (*Trichosurus vulpecula*). We quantified two measures of problem-solving performance: trial outcome (success/failure) and time to solve and tested the influence of a range of potential drivers, including individual traits (personality, body weight, sex, and age), mechanistic behaviors that quantify problem-solving approach (work time, functional behavior time, behavioral diversity, and flexibility), and prior experience with the puzzles. We found that mechanistic behaviors were key drivers of performance. Individuals displaying greater persistence (higher work and functional behavior time) were more likely to solve a food-extraction task on their first attempt. Individuals also solved problems faster if they were more persistent and had lower behavioral flexibility. Personality indirectly affected time to solve one of the three problems by influencing time allocated to functional behaviors. Finally, adults solved the most difficult problem faster than juveniles. Overall, our study provides rare insight into the drivers underlying the problem-solving performance of wild animals. Such insight could be used to improve management strategies and conservation efforts, such as food or bait deployment, tailored to suit the innovative foraging abilities of target individuals in new and changing environments.

## Teaser

Hide Solving problems in accessing food can help animals exploit novel resources. Variations in how, and how well, individuals solve problems may influence their ability to adapt. But, what explains this variation? Testing free-ranging urban brushtail possums with food-extraction problems, we found persistent individuals were most successful, that those with lower behavioral flexibility solved problems faster, and adults were better than juveniles at solving a difficult problem. Personality traits influenced how possums approached problem-solving, indirectly affecting their performance.

## INTRODUCTION

Innovation is often defined as the ability to solve a new problem or invent a new solution for an existing problem (e.g., Kummer and Goodall 1985; Reader and Laland 2003). The capacity and ways in which animals solve problems, both in the wild and when faced with artificial, standardized tasks, are often used as empirical proxies for the ability to innovate (see Griffin and Guez 2014). In the wild, the ability to solve problems can help animals exploit a range of ecological opportunities. For example, greater problem-solving abilities can enable individuals to better exploit novel foods, where new food-extraction techniques are required to access the desired resource. The use of a new food-extraction technique is exemplified by blue tits, *Cyanistes caeruleus*, opening and feeding from milk bottles (Fisher and Hinde 1949; Hinde and Fisher 1951). Such an ability to solve novel problems can also help animals adapt to changing environments and has been linked both to species invasion success (Sol et al. 2002; Tebbich et al. 2010; Chow et al. 2018) and to the ability of wildlife, such as birds (e.g., Audet et al. 2016; Biondi et al. 2021) and mammals (Mazza and Guenther 2021), to thrive in urban environments. Understanding what drives the ability of individuals to innovate may, therefore, aid the design of effective management solutions and conservation efforts, such as targeted pest management or the reduction of human–wildlife conflict by, for example, limiting foraging in waste bins to help reduce the spread of zoonotic and reverse zoonotic diseases.

The ability to solve novel problems can vary among individuals of the same species. Such intra-specific variation has been demonstrated in a wide range of mammals from squirrels (eastern gray, *Sciurus carolinensis*, and Eurasian red squirrels, *Sciurus vulgaris*) (Chow et al. 2018) and common brushtail possums, *Trichosurus vulpecula,* (Wat, Banks, et al. 2020), to mouse lemurs (gray mouse, *Microcebus murinus,* and Madame Berthe’s mouse lemurs, *Microcebus berthae*) (Henke-von der Malsburg and Fichtel 2018) and spotted hyenas, *Crocuta crocuta* (Benson-Amram and Holekamp 2012; Benson-Amram et al. 2013). Intra-specific variation is partly the result of the myriad of factors that could influence an individual’s problem-solving capacity, ranging from state-dependent factors and behavioral traits to environmental conditions and social facilitation (Reader and Laland 2003).

Most drivers affecting inter-individual variation in problem-solving performance fall into two categories: 1) mechanistic behaviors—that is, an individual’s problem-solving approach quantified by the actions displayed when interacting with a problem and 2) individual traits—such as age, size, sex, and personality.

Two examples of mechanistic behaviors that can influence an individual’s problem-solving performance are behavioral diversity and persistence. Greater behavioral (motor) diversity provides an individual with more “raw material” to solve a problem (Griffin and Guez 2014) and can increase the likelihood of success (e.g., Griffin et al. 2014; Daniels et al. 2019). Persistence, that is, time spent engaged with a problem, maybe a generic measure of motivation (see Griffin and Guez 2014) and can also lead to greater success. For example, more persistent squirrels are more likely to extract an attractive food reward from a puzzle box than less persistent individuals (Chow et al. 2016, 2018).

Individual traits, such as age, can affect problem-solving performance both directly and indirectly, via their influence on mechanistic behaviors. Adults are often better problem-solvers than juveniles (see Amici et al. 2019), possibly because with age comes experience and the accumulation of motor skills (e.g., Reader and Laland 2001; Griffin and Guez 2014). In contrast, juveniles are the more innovative in several passerine species as a result of their greater persistence (Morand-Ferron et al. 2011; Griffin et al. 2014).

Personality traits—individual behavioral differences that are consistently exhibited over time or across situations (Reale et al. 2007)—can influence how an individual perceives and interacts with its surroundings (Reale et al. 2007) and can consequently affect an individual’s ability to solve problems (Sih and Del Giudice 2012). As with age, personality traits affect problem-solving both directly and indirectly via their relationship with mechanistic behaviors. For example, in captive trials, more active and explorative African striped mice, *Rhabdomys pumilio*, were more persistent when solving a food-extraction problem (Rochais et al. 2021). Similarly, docility negatively correlated with persistence and behavioral diversity in common brushtail possums attempting a difficult escape box problem (Wat, Banks, et al. 2020).

A final factor affecting problem-solving performance is learning from previous experience. Opportunities to learn can be classified into two groups: 1) generic experience—knowledge or skills gained from a range of prior experiences that can be applied to a novel problem and 2) specific experience—knowledge or skills gained from previous experience with a given problem and applied over repeated attempts. For example, when New Caledonian crows, *Corvus moneduloides*, spontaneously solve a novel meta-tool task, the behaviors they employ suggest the use of generic experience via analogical reasoning (Taylor et al. 2007) (i.e., the mapping of prior task experience onto a similar, yet novel problem; see French 2002). Examining the effect of repeat experiences with a specific task is common in problem-solving research. For example, both guppies, *Poecilia reticulata* (Mair et al. 2021), and common brushtail possums (Wat, Banks, et al. 2020) showed improved problem-solving performance over successive attempts at escape box problems.

Most studies investigating the relationship between personality and problem-solving have used captive animals, both wild-caught and captive-bred. However, the performance of animals in captivity may not reflect the propensity of wild individuals to solve problems. For example, captive hyenas are better able to solve problems than their wild counterparts, a difference explained by reduced neophobia and the greater exploratory behavior of captive individuals (Benson-Amram et al. 2013). In contrast, wild Mexican jays, *Aphelocoma wollweberi*, are faster problem-solvers than captive individuals (McCune et al. 2019). Greater problem-solving capacity in the wild compared with captive individuals may be the result of a range of factors, including heightened vigilance (as suggested by McCune et al. 2019) and the introduction of new stressors in captive environments (Morand-Ferron et al. 2016). Consequently, tests with free-ranging individuals provide a better understanding of the natural behaviors of a species in an ecologically relevant context.

Few studies have investigated the relationship between problem-solving and personality in free-ranging individuals. Of those studies that have, most use a behavior displayed during the problem-solving test as a proxy for a personality trait, confounding the effects of mechanistic behaviors and personality, or focusing on a single personality trait, potentially limiting our insight into the complex relationships between personalities and wider behavioral traits. For example, Harvey and Black (2021) tested whether the ability of wild Steller’s jays, *Cyanocitta stelleri*, to solve a string-pulling task was a function of boldness and exploration (terms commonly defined as personality traits; see Reale et al. 2007). However, both metrics were based on behaviors exhibited during a problem-solving trial rather than measured during an independent test of personality.

Studies of free-ranging individuals with multiple robustly measured personality traits have typically used an escape box problem. For example, more exploratory and more active individual common brushtail possums are better able to solve a novel escape box. Vigilance, as a personality trait, can also have a significant positive effect on problem-solving success (Wat, Banks, et al. 2020). Escape box problems can provide insight into problem-solving performance. But, food-extraction tasks are valuable because they reflect a central ecological challenge faced by individuals in new or changing environments, that is, accessing food. Many examples exist of novel foraging techniques enabling individuals to better exploit, and therefore adapt to, their surroundings. For example, the radiation of Darwin’s finches across the Galápagos is thought to be driven by their ability to develop new foraging techniques and thereby exploit novel food types (e.g., Tebbich et al. 2010). The ability to solve foraging-related problems can therefore be especially valuable during periods of environmental change (e.g., Lefebvre et al. 1997) or habitat expansion (see Lowry et al. 2013; Sol et al. 2013).

We aimed to address the paucity of research on free-ranging individuals, testing the effects of individual traits, including personality and mechanistic behaviors on food-extraction problem-solving performance. We used urban common brushtail possums, *T. vulpecula* (hereafter, “possums”), a medium-sized (2–3 kg), a semi-arboreal marsupial. Possums are generalist folivores and opportunistic foragers (Freeland and Winter 1975; Cowan 1990; How and Hillcox 2000), whose diet breadth and type varies as a function of individual personality, with highly exploratory individuals having a broader diet than less exploratory ones (Herath et al. 2021). Possums thrive in novel environments, having successfully expanded their habitat into both urban Australia (Matthews et al. 2004) and as an invasive species in New Zealand (Nugent et al. 2001). Within urban environments, which frequently present novel foraging opportunities, possums are well known to exploit new resources (e.g., foraging in bins or extracting food from containers). Collectively, this suggests possums possess a high propensity to solve problems, enabling them to exploit novel resources and thus making them an ideal subject for this study.

**Figure 1:**
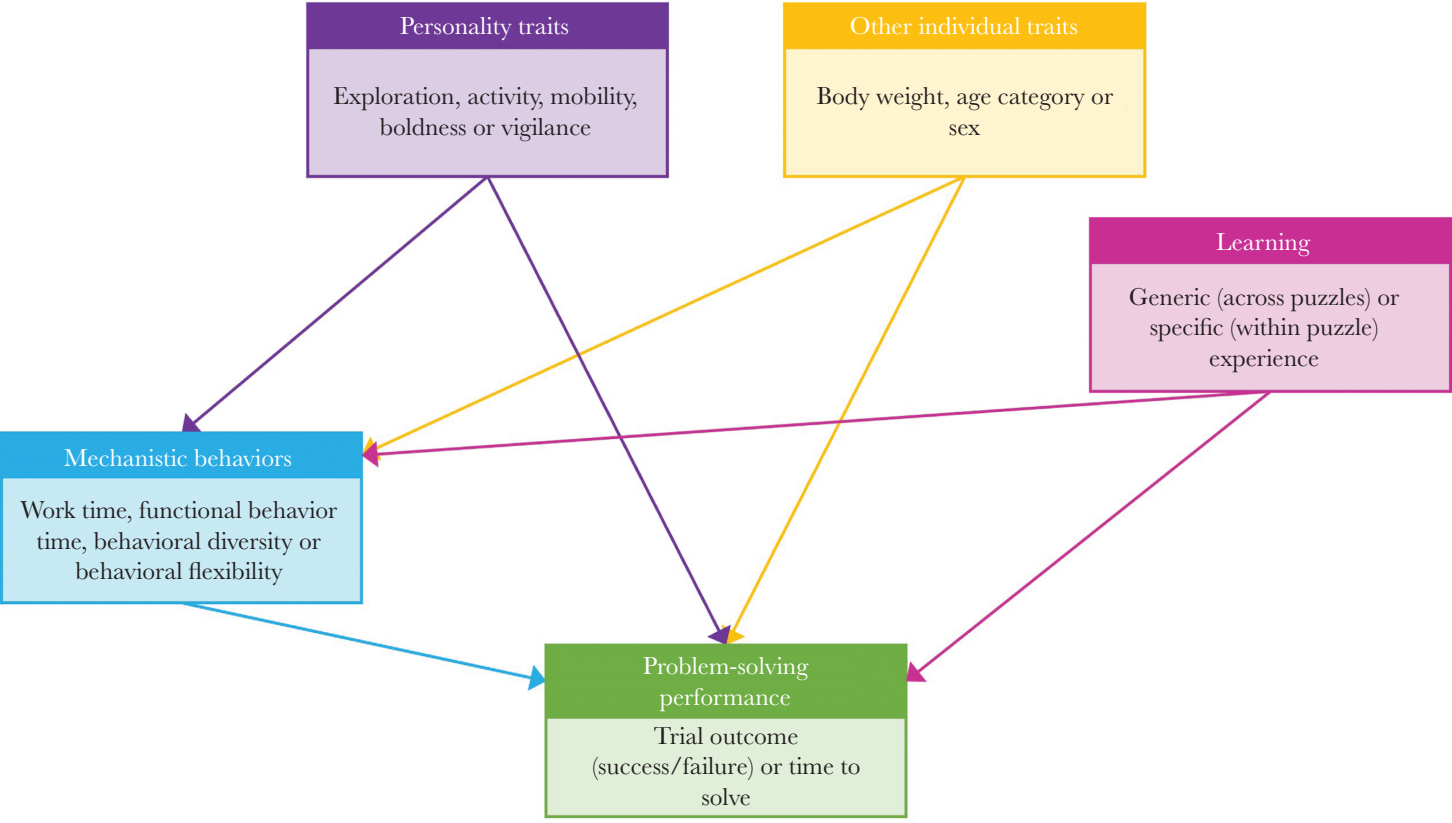
Hypothesized relationships between the drivers of problem-solving performance.

We assessed problem-solving performance across three puzzle types of increasing difficulty. More specifically, we tested whether mechanistic behaviors, individual traits, and experience directly or indirectly affected problem-solving performance (trial outcome [success/failure] and time to solve; Figure 1). For mechanistic behaviors, we quantified work time (i.e., persistence), time spent employing functional behaviors, behavioral diversity, and behavioral flexibility. For individual traits, we considered body weight, age, sex, and a range of personality traits. Lastly, we included two measures of prior experience: generic experience (experience across puzzles) and specific experience (within-puzzle experience). Both individual traits and experience could have a direct or indirect effect on problem-solving performance by acting on the mechanistic behaviors employed during a problem-solving trial.

We expected the mechanistic behaviors displayed during a problem-solving trial to be key predictors of performance, as they reflect an individual’s skill set or level of engagement with a problem. Sex, age, and size were anticipated to influence performance, due to their combined effect on persistence (i.e., motivation to solve), levels of generic experience, and the accumulation of motor skills. For example, adults may be able to use prior knowledge and previously employed motor skills to solve a problem faster. In addition, larger individuals may have a greater capacity to focus their energy on a novel food-extraction problem than their smaller counterparts (as per the Excess Energy Hypothesis (Kummer and Goodall 1985)). We also expected personality traits to affect problem-solving ability either directly or indirectly, via their relationship with mechanistic behaviors. More specifically, we anticipated that the personality traits driving food-extraction problem-solving would be consistent across puzzle types and, therefore, mirror that for escape boxes, with more proactive (more exploratory or more active) individuals being more adept at solving the problems posed (Wat, Banks, et al. 2020). Using the escape box results as our base case, we also expected to see possum problem-solving performance improving over time, that is, to see possums learning from experience (Wat, Banks, et al. 2020).

## MATERIALS AND METHODS

### Study sites and populations

We performed this study at the Camperdown campus of the University of Sydney, Australia (33.89°S, 151.19°E) from November 2018 to January 2019. The 72-hectare university campus is in an urban environment, consisting mainly of private roads and buildings interspersed with gardens, sports grounds, and other green spaces.

We used 15 test sites for the puzzles, distributed a minimum of 20 m apart across the university campus, at sites frequented by possums. This allowed us to record data for 25 uniquely identified possums, 13 females and 12 males (3 of which were juveniles).

### Assessing individual traits

Individuals were trapped using Tomahawk cage traps (63 × 25 × 25 cm), set under large trees and away from likely sources of disturbance, such as walkers and traffic. Traps were set between 19:00 and 20:00 h (i.e., just before sunset), and checked between 22:30 and 05:00 h.

**Figure 2:**
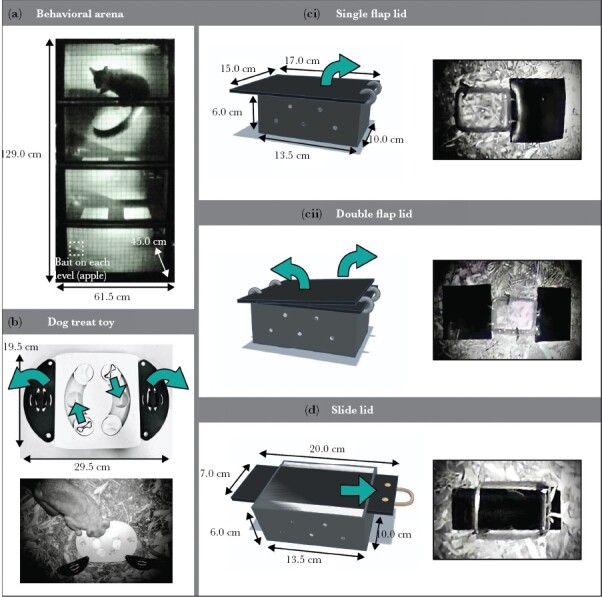
(a) Behavioral arena, baited, and with possum; (b–d) food-extraction problem-solving puzzles: (b) dog teat toy [top] a photograph of a closed puzzle, showing moving parts, and [bottom] a photograph of an open and baited puzzle in the field, with possum; (c) flap lid puzzles [left] a diagram of a closed and [right] a photograph of an open (i) single flap lid and (ii) double flap lid puzzle box; (d) slide lid puzzle [left] a diagram and [right] a photograph of a closed puzzle box. Green arrows show the direction of movement required to open the puzzles. Diagrams created using Tinkercad (© 2023 Autodesk, Inc).

Once captured, individuals were carefully transferred to a handling bag for weighing, then moved to the behavioral arena for personality testing. The arena was an open-field test comprising a module (61.5 × 45.0 × 129.0 cm) with four vertical levels (Figure 2a), each with apple slices (8 g), an appealing food reward, and an infra-red light (following Mella et al. 2016). Possums were filmed in the arena for 5 min using Panasonic HX-A1M infra-red cameras. After the arena test, individuals were returned to the handling bag to determine their sex and, if the first time of capture, to provide with a unique identifier (see below). Once processed, individuals were released back to their trap site.

Quantification of behaviors displayed during the arena test provided five personality measures: exploration, activity, mobility, boldness, and vigilance. Exploration was defined as the time spent in unpreferred levels in the arena (i.e., 5 min minus the maximum time spent on any one of the four levels). Activity was quantified as the total time where any body part was moving (including feeding); and mobility, a subset of activity, as time moving limbs. Vigilance was defined as time spent alert while looking out of the arena. Time spent feeding was used as a proxy for boldness (following Dammhahn and Almeling [2012] and Wilson et al. [1994]). Boldness has been shown to covary with an individual’s physiological stress responses (Baugh et al. 2012; Carere et al. 2010; and for possums A. Raña [BSc(Hons) thesis, 2017]). Of the 25 possums within this study, 16 (64%) had repeat tests (max = 5) in the arena. All behavioral measures were recorded within a long-term dataset of 153 individuals, where 67% had repeat arena test measures, providing robust personality trait indices (following Dingemanse and Dochtermann 2013).

Individual personality trait indices, sex, body weight, and age category (i.e., adult [body weight ≥1.8 kg]/juvenile, hereafter “age”) were collated for all individuals.

### Animal identification

Upon the first capture, possums were given subcutaneous passive integrated transponder tags for accurate long-term, close proximity identification. Small patches of fur were lightly shaved off each possum leaving unique shaving patterns, enabling individuals to be identified on camera in the short term during problem-solving trials.

### Assessing problem-solving performance, mechanistic behaviors, and experience

#### Problem-solving apparatus and procedure

Four of the following problem-solving puzzles were used: a dog treat toy, a single flap lid box, a double flap lid box, and a slide lid box (Figure 2b–d). Each puzzle was baited with sultanas (golden raisins)—a desirable food reward—three in each baited well of the dog treat toy and a total of six in each of the three box puzzles. A puzzle was placed at each testing site. Drilled holes and gaps in the sides of the box puzzles enabled possums to access food odor cues. Tent pegs were used to help fix the puzzles to the ground.

Dog treat toy (Figure 2b)—An Anko Oval Dog Treat Puzzle designed for dogs (supplier Kmart). The 29.5 × 19.5 × 2.5 cm medium-density fiberboard toy contained eight food wells, six of which could be covered at any one time. Throughout the trials, the same four wells were baited and covered (two by small, circular lids and two by large, semi-circular lids hinged by a screw). An individual could push or pull the lids, either along a groove or away from the apparatus, to access the food reward.

Single flap lid (Figure 2ci)—A 13.5 × 10.0 × 6.0 cm plastic box covered by a black, flexible 17.0 × 15.0 × 0.3 cm rubber lid. The lid was hinged to the box by cable ties so that an individual had to lift the lid to extract the food reward. This simple box followed the design of A. Raña (BSc(Hons) thesis, 2017).

Double flap lid (Figure 2cii)—Similar to the single flap lid box, but with two rubber lids (one 14.0 × 15.0 × 0.3 cm, the other 12.0 × 15.0 × 0.3 cm) hinged at opposite sides of the box. Food could be reached in one step (lifting both lids at once, often achieved by an individual pushing its head underneath both lids at the same time) or two steps (lifting one lid at a time). Apparatus design was based on that used by A. Raña (BSc(Hons) thesis, 2017).

Slide lid (Figure 2d)—A 13.5 × 10.0 × 6.0 cm plastic box, covered by a 20.0 × 7.0 × 0.5 cm sheet of black plastic. The plastic lid ran between two thin, parallel, rectangular holes cut into opposite sides of the box. The puzzle was solved by sliding the lid off the box.

Where the soil was too soft to secure the apparatus, the single flap, double flap, and slide lid puzzles could also be solved by flipping the boxes upside down.

Baited dog treat toys and open single-flap boxes were initially deployed at the testing sites for an average of 3 days to habituate possums to the puzzles before testing. We then ran the problem-solving tests over 37 days. Without knowing how well possums would solve each problem, we decided to standardize the order we presented them, with increasing complexity. Testing was run in the order: dog treat toy ➔single flap lid ➔double flap lid ➔slide lid. This approach also allowed us to partially control for prior experience, a hypothesized driver of problem-solving ability. However, we could not fully control for experience as the animals were free-ranging. During testing, each puzzle was left out overnight, with the baited compartments closed.

Visits to the puzzles were recorded by a motion-triggered video camera (Scoutguard SG560k) mounted above the task apparatus. The start of a visit was defined as the time an individual first engaged with a puzzle (i.e., by displaying a behavior directed toward the puzzle, not merely passing it by). The end of the visit was defined as the time of first solve or when an individual ceased to engage with the apparatus, measured as the start of two or more minutes of no engagement. The camera typically recorded a 1-min video but was set to re-trigger immediately, with visits often spanning multiple videos.

Of the recorded visits by known possums, only a subset was considered as suitable trials for our analyses. For inclusion, the puzzle had to be in camera view at the start of the visit, and the required puzzle compartments were closed and baited. Therefore, once a puzzle had been solved, no further visits that night were considered. Overall, 210 visits were classed as trials. Of the remaining 506 visits, the vast majority were possums exploring a puzzle that had been solved earlier that evening.

As a result of testing free-ranging possums, the total number of trials attempted by any one individual varied between 1 and 25 (mean = 9.32). The variety of trials undertaken also differed between individuals (range 1–4 puzzle types, mean = 2.8).

#### Problem-solving measures

We quantified two problem-solving performance measures: trial outcome (success/failure) and time to solve (seconds). Success was defined as an individual reaching into the task apparatus with its head or front paw, or visibly consuming the food reward. Time to solve was measured as the time from the start of a visit to trial success. Where individuals failed to solve a puzzle, no time to solve was recorded. For trials that spanned several videos, an adjustment was made to time to solve to account for any trigger delays between recordings.

**Figure 3:**
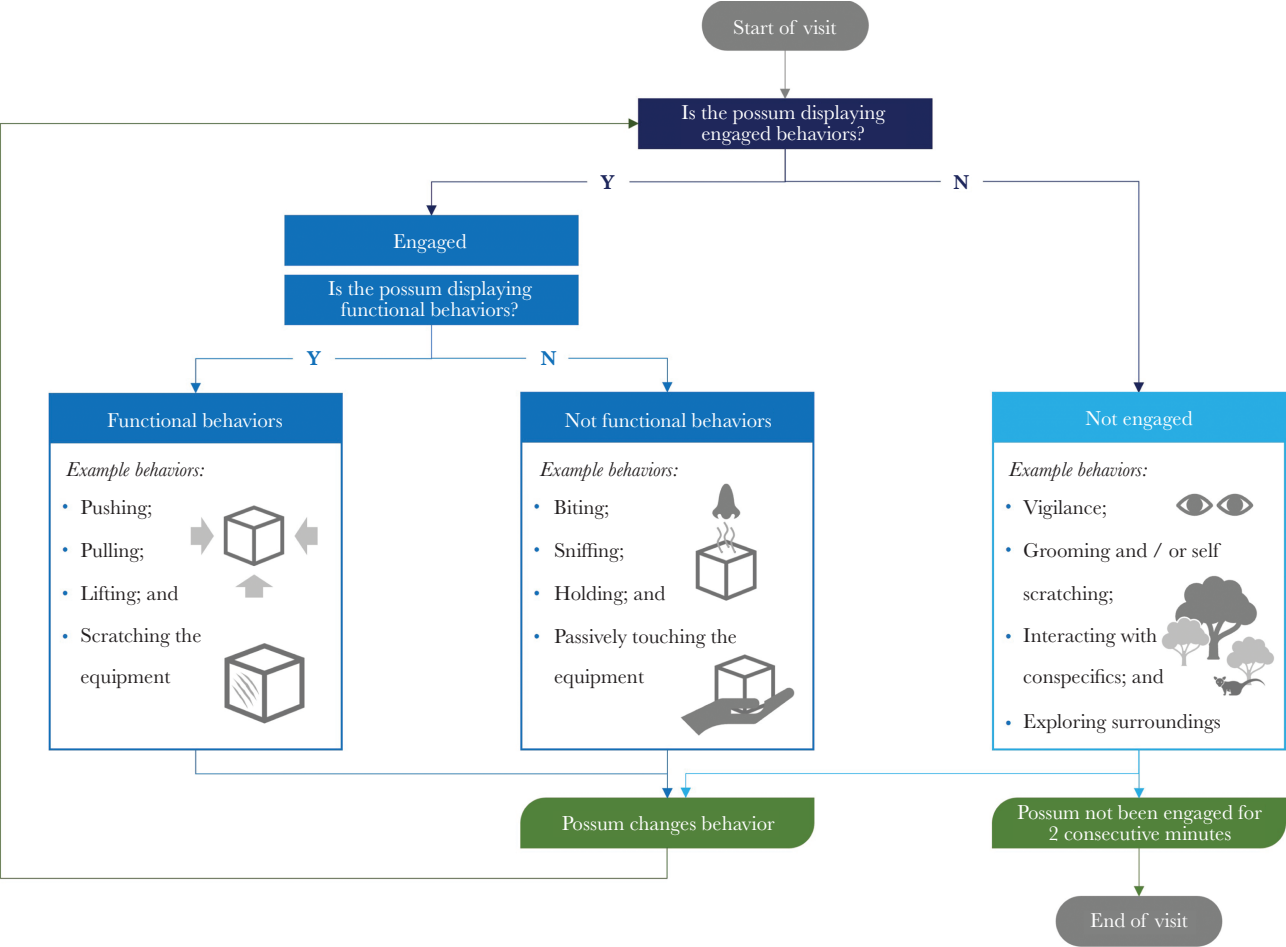
Decision tree to categorize behaviors displayed during a trial (applicable to all puzzles).

Each possum’s actions during a trial were quantified using BORIS (Friard and Gamba 2016). To convert these actions into mechanistic behaviors, we first categorized them into the following three groups: 1) engaged and functional; 2) engaged and not functional; and 3) not engaged (Figure 3). Functional behaviors are often defined as those directed toward a functional element, which, if manipulated, would result in direct access to the reward (e.g., Thornton and Samson 2012; Amici et al. 2020). However, the flap and slide-lid puzzle boxes could be lifted, so a wide range of approaches could be functional. Here, functional behaviors were, therefore, defined as those that, if targeted at the right part of the task apparatus, could lead to trial success. In contrast, engaged and not functional behaviors were those that, although targeted toward a puzzle, could not directly result in an individual accessing the food reward. Such behaviors included sniffing, biting, passively touching, and holding the apparatus. When displaying any behavior not directed toward the puzzle, individuals were not considered to be engaged. Not-engaged behaviors included vigilance, grooming, interacting with conspecifics, and exploring the area. By categorizing the data in this way, we extracted the following measures of an individual’s mechanistic behavior from the dataset: work time, functional behavior time, behavioral diversity, and behavioral flexibility.

##### Work time.

A proxy for persistence (following e.g., Benson-Amram and Holekamp 2012; Benson-Amram et al. 2013). The proportion of time spent displaying engaged behaviors.

##### Functional behavior time.

A subset of work time. The proportion of total time engaged spent displaying functional behaviors (following Wat, Banks, et al. 2020).

##### Behavioral diversity.

This metric considers the range of motor skills used (Griffin and Guez 2014) and is often found to influence problem-solving performance (e.g., Griffin et al. 2014; Daniels et al. 2019). Here, we counted the number of different engaged behaviors exhibited during a trial.

##### Behavioral flexibility.

Flexibility measures the rate of switching between engaged behaviors (e.g., Chow et al. 2016, 2018). It was calculated by dividing the number of times an individual changed between engaged behaviors by the total time engaged.

Lastly, we quantified experience. The experience influenced the possum problem-solving ability in the escape box test (Wat, Banks, et al. 2020), with time to solve decreasing in later attempts (i.e., with increasing attempt number). Here, we measured experience in the following two ways:

Generic experience, that is, experience across puzzles—The cumulative number of previous visits (i.e., any visit in which a possum engages with a puzzle, whether the puzzle had yet to be, or had already been, solved) across all puzzle types. For an individual attempting their first slide lid trial, for example, this measure included all prior visits to the dog treat toy, single flap lid, and double flap lid puzzles.

Specific experience, that is, within-puzzle experience—Due to the high first trial success rates (see below), we opted to exclude trial outcome (success/failure) from this analysis. Therefore, for each puzzle type, we investigated whether previous experience with that puzzle affected the time to solve over consecutive successive trials (first vs. second successful attempts only). If a possum failed the puzzle between their two successful attempts, they were excluded from the analysis.

Of the 210 trials, we were able to determine the outcome (i.e., whether the trial was a success or failure) for 207. We were then able to quantify a complete set of problem-solving data, including time to solve and mechanistic behaviors, for 161. Any missing data for trial outcome, time to solve, or a mechanistic behavior, was a result of either camera malfunctions or possums moving the puzzle temporarily out of view.

### Statistical analysis

#### Drivers of trial performance

To help control for experience, when considering trial outcome (success/failure), only an individual’s first trial with each puzzle type (i.e., the first instance an individual engaged with a baited and closed puzzle, in camera view) was included in the following analyses. Similarly, when assessing the drivers of time to solve, only an individual’s first successful attempt at each puzzle type was included. However, as possums solved both the single and double flap lid trials in essentially the same way, we chose to combine these results. The first trials for the combined “flap lid” puzzle type, therefore, included an individual’s first trial at the single flap lid puzzle or, if they did not attempt the single flap lid, their first trial at the double flap lid puzzle.

The relationship between sex and body weight of adult possums was significant (Anova *F*(1, 19) = 4.78, *P* = 0.04), but as all juveniles in the study were male, we opted to exclude sex from the following analyses. Some personality traits (boldness, activity, and exploration) form a proactive–reactive syndrome (Wat, Banks, et al. 2020). However, we considered the traits separately because they captured distinct behaviors that were known to have different influences on the ecology of individuals (e.g., on home range size [Wat, Herath, et al. 2020] and their diet [Herath et al. 2021]).

We analyzed the factors affecting problem-solving performance in two stages. First, we investigated the various drivers of the first trial outcome (success/failure). Second, we tested the relationships between the direct and indirect drivers of time to first solve (for successful attempts only) using a structural equation modeling (SEM) approach.

#### Drivers of first trial outcome (success/failure)

Here, we used first trials with complete data for the flap and slide lid puzzles (success rates 73% and 72%, respectively). We excluded the dog treat puzzle because its success rate was so high (91%; 10/11 individuals succeeded). Data included 33 observations: 15 individuals attempting the flap lid puzzle and 18 attempting the slide lid puzzle. For behavioral diversity, we used the proportion of available engaged behaviors displayed, rather than the count because the total maximum count differed between the two puzzle types.

Due to the low number of failures, we were unable to statistically consider multiple drivers within a single model. Therefore, we tested the direct effects of the various drivers on the first trial outcome (success/failure) in separate models (following Wat, Banks, et al. 2020). We considered the effects of mechanistic behaviors (work time, functional behavior time, behavioral diversity, and behavioral flexibility), individual traits (age, body weight, personality), and generic experience. We fitted a series of generalized linear mixed-effect models with binomial distribution and logit link using R package lme4 (Bates et al. 2015), with possum ID included as a random effect. Puzzle type had no effect on the trial outcome, so it was excluded from this analysis. The inclusion of age within the model resulted in complete separation, with all juveniles successfully completing their first trial. Consequently, age was also excluded from this analysis.

We then examined the drivers of any significant mechanistic behaviors (identified via the above analysis). We considered personality, other individual traits, and generic experience. We ran linear mixed-effect models with Gaussian distribution and Possum ID as a random effect, using lme4 (Bates et al. 2015). Each model included a single driver, again following Wat, Banks, et al. (2020). Where puzzle type had a significant effect on the mechanistic behaviors, puzzle type was included in the model both as a main effect and as an interaction with the driver of interest. Models were tested for normality and homogeneity of variance, and where required, the response variable was log-transformed to satisfy these assumptions.

#### Drivers of time to first solve (SEM analysis)

We analyzed 33 trials in which an individual first solved a given puzzle (dog treat toy *n* = 10, flap lid *n* = 11, and slide lid *n* = 12 (excluding an outlier)). One individual, an adult possum within the slide lid data, spent a total of 93 s displaying vigilant behaviors (vs. a mean of 15) and, therefore, not engaged in the problem. It was considered an outlier and excluded from the following analysis.

To test the effect of puzzle type on time to first solve, we first ran a linear mixed-effect model with a Gaussian distribution and possum ID included as a random effect. Time to solve was log-transformed to satisfy normality and homogeneity of variance assumptions.

For each puzzle type, we then analyzed the underlying drivers of time to first solve via an SEM approach, using R package PiecewiseSEM (Lefcheck 2016). We used an SEM approach as it allowed for the multivariate analysis of complex hypothesized systems (such as our hypothesized model in Figure 1). It also enabled us to assess both the direct and indirect effects of explanatory variables on trial performance in one model. This capacity was particularly valuable for individual traits (personality, body weight, and age) and generic experience, as these could influence problem-solving ability both directly and indirectly through their effect on mechanistic behaviors.

We used Figure 1 as the starting hypothesis for each SEM. Each SEM consisted of three linear models with Gaussian distributions, one tested the drivers of time to first solve, and the remainder assessed the drivers of two out of the four available mechanistic behaviors (work time, functional behavior time, behavioral diversity, or behavioral flexibility). Highly correlated mechanistic behaviors (*R* ≥ 0.7) were not included in the same SEM. Typically, all three linear models were based on the same explanatory variables: body weight or age, a single personality trait, and generic experience, with the models for time to first solve also including the two selected mechanistic behaviors. However, we opted to exclude generic experience as an explanatory variable for the dog treat toy, as individuals had no (or very limited) experience with any puzzle apparatus at this time.

As body weight might be explained, at least in part, by the age of the individual, body weight and age were not included in the same SEM. We also opted to test a single personality trait at a time (following Wat, Banks, et al. 2020, Wat, Herath, et al. 2020; Rochais et al. 2021). This approach enabled us to identify the most informative personality trait for any given problem-solving scenario. Finally, to limit the number of explanatory variables tested in a single model, we included only two of the four available mechanistic behaviors within an SEM.

Models were checked for normality and homogeneity of variance, and where required, the response variable was log-transformed to satisfy these assumptions. Models with a Fisher’s *C P*-value ≤ 0.05 were rejected. Of the remaining model options, model fit was compared using Fisher’s *C* and Akaike information criterion values (following Lefcheck 2016). The output of this analysis was a single model, which best reproduced the data and highlighted the key interrelated drivers explaining time to first solve.

The same approach was repeated for each puzzle type. We identified the most parsimonious model for each puzzle type rather than testing them all together because 1) each possum had a unique combination of prior trials; 2) each puzzle type required different approaches to solve it; 3) task complexity likely varied among puzzles; and 4) there were constraints posed by the selected SEM R package (e.g., the inability to account for random effects in multigroup analysis).

#### Evidence of within-puzzle learning

We focused on change in time to solve from the first to second trials to determine whether an individual’s performance improved with (i.e., individuals learned from) specific (within-puzzle) experience. Again, only successful trials with complete data were used. In addition, only individuals with first and second successful trials (flap lid *n* = 8, and slide lid *n* = 10 [excluding the outlier]) were included in this analysis. The dog treat toy puzzle was dropped due to sample size constraints.

For the flap and slide lid puzzle types, we tested whether the time to solve was affected by the number of previously successful trials. We ran linear mixed-effect models with a Gaussian distribution, possum ID as a random effect, and successful attempt number as the explanatory variable. Models were checked for normality and homogeneity of variance, and the response variable was either square root or log-transformed as required.

## RESULTS

### Drivers of first trial performance

#### Drivers of first trial outcome (success/failure)

**Figure 4:**
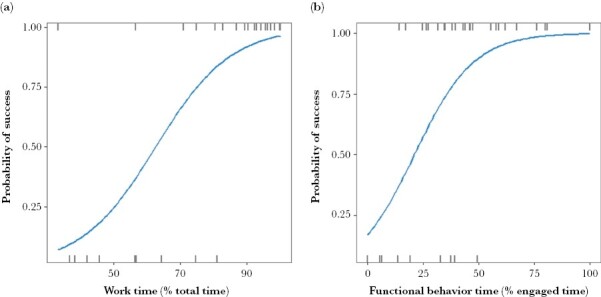
Direct significant relationships (Supplementary Table 1) showing the effect of (a) work time (proportion of time spent engaged in the puzzle), and (b) functional behavior time (proportion of time engaged in displaying functional behaviors) on the first trial outcome (success/failure) for the flap (*n* = 15) and slide lid (*n* = 18) puzzles.

Two mechanistic behaviors had a significant effect on the probability of success: work time (χ^2^(1, 33) = 9.41, *P* = 0.002) and functional behavior time (χ^2^(1, 33) = 6.17, *P* = 0.013; Supplementary Table 1). Individuals were more likely to solve their first trial with greater work time (proportion of total time spent engaged) (Figure 4a) and functional behavior time (proportion of time engaged spent displaying functional behaviors) (Figure 4b). No individual trait (personality, body weight, age) or generic experience had a significant effect on the probability of success.

**Figure 5:**
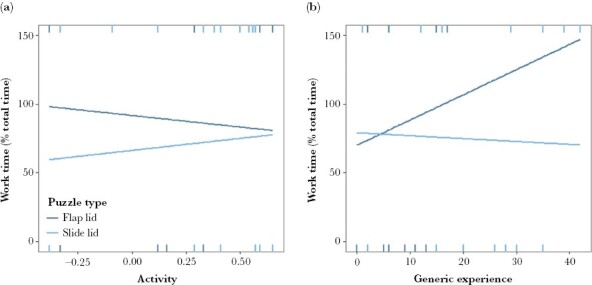
Significant relationships (Supplementary Table 2) showing the effect of (a) activity (personality trait) and (b) generic experience (number of previous visits across puzzle types) on work time (proportion of time spent engaged in the problem posed) for an individual’s first attempt at the flap (dark blue line, *n* = 15) and slide lid (light blue line, *n* = 18) puzzles.

As a main effect, only puzzle type had a significant effect on work time, with none of the individual traits (personality, body weight, and age) or generic experience having a significant influence on this mechanistic behavior (Supplementary Table 2). However, activity (personality trait) had a significant effect on work time as an interaction with puzzle type (χ^2^(1, 33) = 7.51, *P* = 0.006), with greater activity increasing work time for the slide lid puzzle and reducing work time for the flap lid puzzle (Figure 5a). Similarly, exploration and vigilance had significant effects as an interaction with puzzle type, with their directional effect mirroring that for activity (Supplementary Table 2). The interaction between generic experience and puzzle type also had a significant effect on work time (χ^2^(1, 33) = 9.85, *P* = 0.002), with work time slightly decreasing with generic experience for the slide lid puzzle but increasing for the flap lid puzzle (Figure 5b).

As puzzle type did not have a significant effect on functional behavior time, it was excluded from this analysis. None of the individual traits (personality, body weight, and age) or generic experience significantly influenced functional behavior time (Supplementary Table 3).

#### Drivers of time to first solve (SEM analysis)

**Figure 6:**
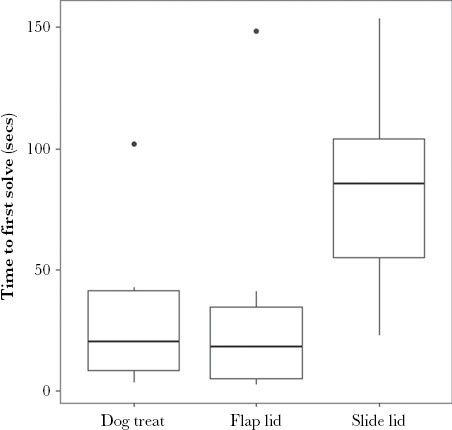
Time to first solve by puzzle type (dog treat toy *n* = 10, flap lid *n* = 11, and slide lid *n* = 12).

Time to first solve (log-transformed) varied with puzzle type (χ^2^ (2, 33) = 24.03, *P* <0.001), with the slide lid puzzle taking significantly longer to solve than either the dog treat toy or flap lid puzzles (Figure 6).

##### Dog treat toy.

**Figure 7:**
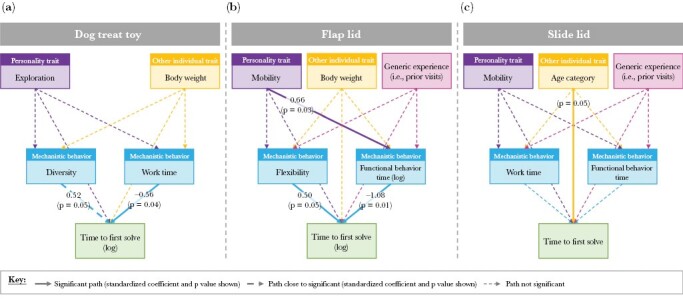
Best-fitting SEM models for (a) dog treat toy (*n* = 10); (b) flap lid (*n* = 11); and (c) slide lid (*n* = 12) puzzles. SEMs show links between personality traits, other individual traits (body weight or age), generic experience (flap lid and slide lid puzzles only), mechanistic behaviors (work time, functional behavior time, behavioral diversity, or behavioral flexibility), and problem-solving performance (time to solve) of first successful trials. Wide solid lines indicate path significance (*P* < 0.05), wide hatched lines indicate the path is close to significant (and therefore worthy of note), and thin hatched lines show all other paths tested in the model. For significant paths and paths of note, standardized coefficients and *P* values are shown.

**Figure 8:**
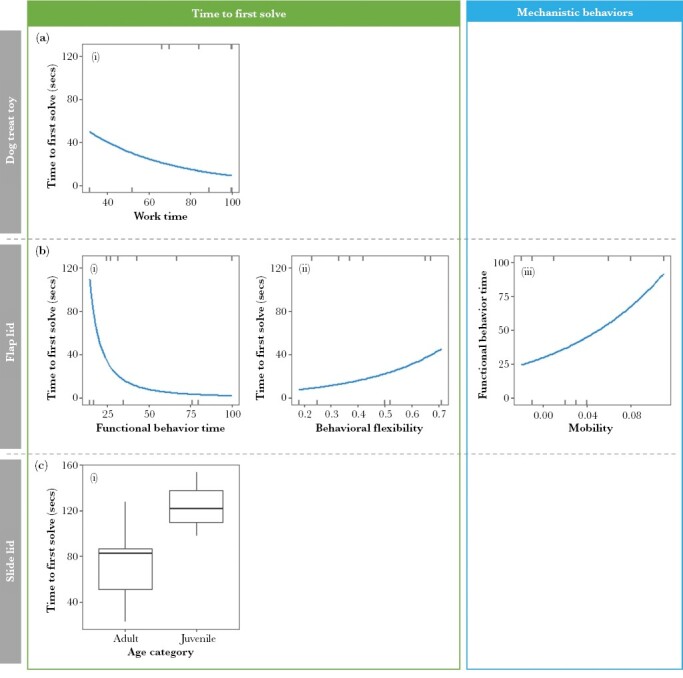
Significant relationships (Table 1, Figure 7) between personality traits, other individual traits (body weight or age), generic experience (flap lid and slide lid puzzles only), mechanistic behaviors (work time, functional behavior time, behavioral diversity, or behavioral flexibility), and problem-solving performance (time to first solve). The significant drivers of time to first solve for each puzzle type include (a) Dog treat toy (*n* = 10): (i) work time; (b) Flap lid (*n* = 12): (i) functional behavior time, (ii) behavioral flexibility, and (iii) mobility via its effect on functional behavior time; and (c) Slide lid (*n* = 11): (i) age.

The best-fitting SEM model included the explanatory variables behavioral diversity and work time, body weight, and exploration (personality trait) (Table 1a, Figures 7a and 8a). Behavioral diversity and work time had the greatest influence, with a higher work time significantly reducing time to first solve (standardized β = −0.56) and a greater behavioral diversity increasing problem-solving time (0.52). Neither body weight nor exploration was important.

**Table 1:** Complete SEM output, including the best-fitting SEM models for (a) dog treat toy (*n* = 10); (b) flap lid (*n* = 11); and (c) slide lid (*n* = 12) puzzles

Puzzle type	Response variable	Driver	df	*P*	Std. estimate
(a) Dog treat toy	Work time	Body weight	7	0.637	0.18
		Exploration	7	0.804	−0.10
	Behavioral diversity	Body weight	7	0.869	0.06
		Exploration	7	0.547	−0.23
	Trial duration (secs) (log)	Body weight	5	0.242	−0.27
		Exploration	5	0.177	−0.32
		Work time	5	**0.041**	−0.56
		Behavioral diversity	5	0.053	0.52
(b) Flap lid	Behavioral flexibility	Body weight	7	0.668	0.17
		Mobility	7	0.410	0.31
		Generic experience	7	0.637	−0.18
	Functional behavior time (log)	Body weight	7	0.119	0.45
		Mobility	7	**0.030**	0.66
		Generic experience	7	0.623	−0.13
	Trial duration (secs) (log)	Body weight	5	0.342	0.23
		Mobility	5	0.850	0.05
		Generic experience	5	0.386	−0.18
		Behavioral flexibility	5	**0.048**	0.50
		Functional behavior time (log)	5	**0.012**	−1.08
(c) Slide lid	Work time	Age category	8	0.619	-
		Age [Adult]	8	**<0.001**	-
		Age [Juvenile]	1	**<0.001**	-
		Mobility	8	0.307	−0.38
		Generic experience	8	0.246	−0.38
	Functional behavior time	Age category	1	0.229	-
		Age [Adult]	8	**0.001**	-
		Age [Juvenile]	8	**<0.001**	-
		Mobility	8	0.285	−0.37
		Generic experience	8	0.647	0.14
	Trial duration (secs)	Age category	1	**0.047**	-
		Age [Adult]	6	**0.001**	-
		Age [Juvenile]	6	**0.001**	-
		Mobility	6	0.485	0.25
		Generic experience	6	0.821	−0.07
		Work time	6	0.402	−0.27
		Functional behavior time	6	0.175	0.49

SEMs show links between personality traits, other individual traits (body weight or age), generic experience (flap lid and slide lid puzzles only), mechanistic behaviors (work time, functional behavior time, behavioral diversity, or behavioral flexibility), and problem-solving performance (time to solve) of first successful trials. Bold indicates statistical significance (*P* < 0.05).

##### Flap lid.

The best-fitting SEM model included functional behavior time and behavioral flexibility, body weight, mobility (personality trait), and generic experience (Table 1b, Figures 7b and 8b). Only functional behavior time and behavioral flexibility had a significant direct effect on the time to first solve. The greater an individual’s behavioral flexibility, the longer they took to solve the puzzle (standardized β = 0.50). In contrast, the greater their functional behavior time, the quicker they solved the puzzle (−1.08). Personality had a significant indirect effect on time to solve, with more mobile individuals demonstrating greater functional behavior time (0.66).

##### Slide lid.

The best-fitting SEM model included work time and functional behavior time, age (rather than body weight), mobility, and generic experience, (Table 1c, Figures 7c and 8c). Only age was significant, with adult individuals solving this puzzle faster than juveniles.

### Evidence of within-puzzle learning

There was no significant effect of successful attempt number (first to second consecutive successful attempts only) on time to solve across both flap and slide lid puzzle types (Table 2).

**Table 2:** Analysis of deviance table (Type II Wald chi-square tests) for linear models on time to solve by successful attempt number

Puzzle type	LRΧ^2^	df	*P*
Flap lid (log)	1.58	1	0.209
Slide lid (sqrt)	0.62	1	0.430

The first two successful attempts are considered for flap (*n* = 8) and slide lid puzzles only (*n* = 10).

## DISCUSSION

We investigated the effect of a wide range of drivers on the food-extraction problem-solving performance of free-ranging urban possums. Our results support elements of our hypothesized problem-solving system (Figure 1). We found work time, functional behavior time, behavioral flexibility, and, to a lesser extent, behavioral diversity were strong drivers of performance. Work time positively affected both trial outcome (Figure 4a) and problem-solving speed (Table 1a, Figures 7a and 8ai), suggesting that greater persistence aided problem-solving performance. Functional behavior time—the time spent using functional behaviors as a proportion of the time spent engaged in the puzzle—could be considered a form of proactive or directed persistence. As with work time, individuals employing greater functional behavior time were more likely to solve their first trial (Figure 4b) and solve faster (Table 1b, Figures 7b and 8bi). In summary, the greater the proportion of time an individual invested in proactively manipulating a problem, the better their problem-solving performance. These findings are similar to results considering the food-extraction abilities of free-ranging squirrels where both persistence (measured as attempt rate) and an equivalent of functional behavior time (proportion of behaviors that were effective) were significant predictors of time to solve (Chow et al. 2018).

The SEM model for the flap lid puzzle also demonstrates the role of behavioral flexibility (Table 1b, Figures 7b and 8bii). Individuals switching at a greater rate between functional behaviors took longer to solve the problem. However, behavioral flexibility did not affect the likelihood of success. These findings are at odds with many studies, in which greater behavioral flexibility was associated with greater problem-solving performance (e.g., in Indian mynas, *Acridotheres tristis* [Griffin and Diquelou 2015] and Caracara Chimango, *Milvago chimango* [Biondi et al. 2021]) but consistent with others (e.g., in gray squirrels, where greater behavioral flexibility led to decreased persistence and functional behavior time [Chow et al. 2016]). Given the best solution for the flap lid puzzle was to simply lift the lid, trying alternative solutions presumably just wasted time. The impact of behavioral flexibility may therefore depend on the nature of the problem posed and steps required to solve it.

Behavioral diversity, that is, the range of motor skills displayed when attempting to solve a task, was also an important component of possum problem-solving performance. Although it did not affect the likelihood of success, greater behavioral diversity increased the time to solve the dog treat toy (Table 1a, Figure 7a). Again this finding is at odds with many previous studies (see Griffin and Guez 2014). However, the negative relationship found here may have arisen because individuals taking longer had more time to try a broader range of motor skills. Alternatively, the more behaviors employed means the less an individual may have been persevering with a more effective approach. As with behavioral flexibility, an increase in behavioral diversity may actually impede problem-solving performance, when a single solution (sliding a lid) best solved this relatively simple puzzle. With more complicated or multi-solution puzzles, such as a multi-access box, the relationship between behavioral diversity and problem-solving performance may be the opposite, that is, positive, as found with racoons*, Procyon lotor* (Daniels et al. 2019) and Indian mynas, *Sturnus tristis* (Griffin et al. 2014).

We also found that age influenced success for the slide lid puzzle (Table 1c, Figures 7c and 8ci), which, based on time to solve, was the most complex of the three problems posed (Figure 6). Adults were the more proficient problem-solvers, as found in other studies (see Amici et al. 2019). This relationship may arise from an association between age and generic problem-solving experience, which may become more important for complex problems. Alternatively, larger (adult) individuals may simply manipulate the puzzle more easily.

An individual’s personality also affected problem-solving performance, albeit indirectly. For the flap lid puzzle, the personality trait “mobility” influenced speed of success (Table 1b, Figures 7b and 8biii) via its relationship with the mechanistic behaviors employed. More mobile individuals spent a greater proportion of time engaging functional behaviors, which, in turn, improved problem-solving speed. This indirect relationship indicates that personality affects approach rather than performance. A similar result was found in captive-born bank voles, *Myodes glareolus*, where more active individuals demonstrated greater persistence (measured as latency to correct a decision) when faced with maze tasks (Mazza et al. 2018).

That said, direct effects of personality on problem-solving performance have been found for other types of problems. For example, in possums, escape box success increases with both vigilance and activity personality traits, with only highly explorative individuals able to solve the more complex of escape box puzzles (Wat, Banks, et al. 2020). Studies of rodents have also shown similar discrepancies between results from different trial designs (Rowell and Rymer 2020; Rochais et al. 2021). Collectively, such findings highlight the importance of task type and context on an individual’s problem-solving performance. One simple explanation for this is that the behaviors, or movements, required to solve a problem may vary by task type. Movements to explore and solve an escape box may be more strongly aligned with personality traits, for example, than those for foraging tasks.

An alternative explanation for such variation in the drivers of problem-solving performance considers differences in motivation. An individual’s willingness to engage with and solve a food-extraction task may be the result of a range of factors, including hunger (e.g., Cooke et al. 2021), perceived risk (e.g., Eccard et al. 2020), and curiosity (as seen in captive Bornean, *Pongo pygmaeus,* and Sumatran orangutans, *Pongo abelii* (Damerius et al. 2017)). In contrast, the need to escape may be a much stronger and more universal motivator. Of the possums that did not successfully complete their first trial, most (60%) were vigilant within the last second of their attempt. This result suggests that trial failure arose due to possums reprioritizing motivations, from foraging to fleeing, rather than an inability to solve the problem. Such opposing motivational drivers suggest the potential costs (e.g., lack of vigilance and thus higher predation risk) of advanced problem-solving, with the resulting cost-benefit trade-off possibly helping maintain variation in problem-solving performance in wild populations. The potential importance of trade-offs in motivation when problem-solving was highlighted by van Horik and Madden (2016), who found that variation in the problem-solving ability of captive-reared pheasants, *Phasianus colchicus*, could be explained by noncognitive motivational mechanisms alone. To better understand the role motivation plays in problem-solving success, it helps to measure, standardize, or manipulate an individual’s motivation. This is difficult with free-ranging individuals, but the inclusion of proxy measures of motivation, such as body condition (as used by, e.g., Benson-Amram and Holekamp 2012; Henke-von der Malsburg and Fichtel 2018), when assessing problem-solving performance may be useful.

Lastly, differences in task complexity may also help explain the variation in key drivers of performance between task types (i.e., food-extraction vs. escape box). Within our study, the high first-trial success rate (≥72%) indicates all tasks had relatively simple solutions. Such task simplicity left individuals with limited scope for improvement, which may partly explain the lack of improvement in problem-solving performance over time. Possums’ performance may also improve after the first two successful trials, but this was beyond our scope to test. In contrast, we know possums can learn from specific experiences with escape box puzzles (Wat, Banks, et al. 2020). This suggests that for simple food-extraction problems, individuals may be repeatedly employing a trial-and-error approach. Consistent with this, individuals using greater behavioral diversity and flexibility took longer to first solve. Hence, these individuals may be simply trying any available approach until they happen upon a solution. Employing a trial-and-error approach during a first attempt at a novel problem seems to be common, for example, wild baboons, *Papio anubis*, attempting a string-pulling task (Laidre 2008). More complex problems may therefore better reveal relationships between some of our hypothesized drivers, such as personality traits, and food-extraction problem-solving ability.

Similarly, a difference in task complexity may explain, at least in part, the change in the relative importance of problem-solving drivers across our three food-extraction puzzles. This variation is best highlighted by comparing the three best-fitting SEM models (Table 1, Figure 7) and the relationships between the drivers of work time and puzzle type (flap and slide lid only) during a possum’s first trial (Figure 5). The potential importance of puzzle complexity is suggested by the step change in time to solve between the slide lid problem and previous puzzle types (Figure 6), indicating a marked increase in task difficulty. The changing effect of drivers with puzzle complexity found here would align with findings from a number of problem-solving studies. For example, Chang et al. (2018) presented spiders, *Portia labiate*, with both a simple and difficult lab-based maze problem. Aggressive traits aided accurate decision-making during the simple task; in contrast, docile traits proved more valuable during the difficult problem.

To better understand whether predictable relationships exist between food-extraction performance and its underlying drivers, as well as the impact of both puzzle type and complexity, further research with free-ranging individuals is required. To help unpick these relationships, such work should continue to enhance task complexity and incorporate a means to account for motivation. Through further testing, we should be able to identify, and subsequently manipulate, the key drivers which consistently impact important problem-solving performance.

Enhancing our understanding of how food-extraction problem-solving performance is affected by inter-individual variation and context is valuable for both fundamental and applied reasons. For example, such knowledge may enable us to ascertain whether some individuals are better able than others to expand into, and thrive within, novel environments. Where individuals have successfully adapted to urban environments, we may also be able to use our growing knowledge of problem-solving drivers to identify cohorts at risk of human–wildlife conflict, whether that be via unwelcome foraging behaviors or the spread of zoonotic diseases. For example, bold and explorative individuals often exhibit greater problem-solving abilities (see Amici et al. 2019). These personality traits have also been linked to a lower fear of humans (e.g., Biro and Post 2008) and home ranges spanning both rural and urban areas (Wat, Herath, et al. 2020), with the potential result that bold and explorative individuals are more likely to stoke human–wildlife conflicts. The collective implications of such differences in individual traits or behaviors should therefore be considered when designing conservation and management efforts to ensure outcomes are desirable (see Garvey et al. 2020; Martínez-Abraín et al. 2022). By accounting for inter-individual variation in food-extraction problem-solving capacity, and its underlying drivers, it may be possible to, for example, tailor access to bait stations to target high-risk cohorts (e.g., those posing a greater threat of zoonoses) or design waste management solutions that a wide range of individuals (i.e., not just the average) cannot solve. Overall, there is likely to be substantial value in a robust understanding of the drivers of problem-solving performance.

## Supplementary Material

arad054_suppl_Supplementary_Tables

## Data Availability

Analyses reported in this article can be reproduced using the data provided by Harris et al. (2023).

## Notes

Work was conducted under the approval of the University of Sydney Animal Ethics Committee (AEC Permit 2018/1416) and the New South Wales Department of Planning and Environment (formerly the Office of Environment and Heritage) (Scientific License No. SL102186).
