# Supplementary material for: Grow up, be persistent, and stay focused: keys for solving foraging problems by free-ranging possums
Source: Behav Ecol. 2023 Jun 28;34(5):790–803. doi: 10.1093/beheco/arad054 (PMC10690113; doi:10.1093/beheco/arad054)
Supplement: arad054_suppl_Supplementary_Tables [file arad054_suppl_supplementary_tables.docx]

Supplementary materials

Supplementary materials - Table 1) For the flap (n = 15) and slide lid (n = 18) puzzles: analysis of deviance table (Type II Wald chi-square tests) for generalized linear mixed-effects models on trial outcome (success/ failure) (with possum ID as a random effect). Bold indicates statistical significance (P < 0.05).

| *Driver category* | *Driver* | *LR Χ^2^_1_* | *P* |
| --- | --- | --- | --- |
| Mechanistic behavior | Work time | 9.41 | **0.002** |
|  | Functional behavior time | 6.17 | **0.013** |
|  | Behavioral diversity | 2.53 | 0.112 |
|  | Behavioral flexibility | 1.79 | 0.180 |
| Personality | Exploration | 0.00 | 0.945 |
|  | Boldness | 1.49 | 0.222 |
|  | Activity | 0.32 | 0.571 |
|  | Mobility | 2.42 | 0.120 |
|  | Vigilance | 1.20 | 0.273 |
| Other individual traits | Body weight | 0.00 | 0.972 |
| Generic experience |  | 0.39 | 0.534 |

Supplementary materials - Table 2) For the flap (n = 15) and slide lid (n = 18): analysis of deviance table (Type II Wald chi-square tests) for linear mixed-effects models on work time (with possum ID as a random effect). * Indicates where a response variable has been log transformed. Bold indicates statistical significance (P < 0.05).

|  | *Driver category* | *Driver* | *Driver* | | *Puzzle Type* | | *Driver * Puzzle Type Interaction* | |
| --- | --- | --- | --- | --- | --- | --- | --- | --- |
|  |  |  | *LR Χ^2^_1_* | *P* | *LR Χ^2^_1_* | *P* | *LR Χ^2^_1_* | *P* |
|  | Personality | Exploration | 0.05 | 0.818 | 10.79 | **0.001** | 4.77 | **0.029** |
|  |  | Boldness | 0.00 | 0.953 | 5.50 | **0.019** | 0.14 | 0.711 |
|  |  | Activity | 0.05 | 0.831 | 12.91 | **<0.001** | 7.51 | **0.006** |
|  |  | Mobility | 1.25 | 0.264 | 4.90 | **0.027** | 0.41 | 0.523 |
|  |  | Vigilance* | 1.70 | 0.193 | 6.56 | **0.010** | 5.69 | **0.017** |
|  | Other individual traits | Body weight | 0.02 | 0.881 | 5.25 | **0.022** | 0.11 | 0.740 |
|  |  | Age category | 1.33 | 0.249 | 6.07 | **0.014** | 0.11 | 0.744 |
|  | Generic experience |  | 0.19 | 0.667 | 3.15 | 0.076 | 9.85 | **0.002** |

Supplementary materials - Table 3) For the flap (n = 15) and slide lid (n = 18): analysis of deviance table (Type II Wald chi-square tests) for linear mixed-effects models on functional behavior time (with possum ID as a random effect).

| *Driver category* | *Driver* | *LR Χ^2^_1_* | *P* |
| --- | --- | --- | --- |
| Personality | Exploration | 0.34 | 0.561 |
|  | Boldness | 0.77 | 0.381 |
|  | Activity | 1.86 | 0.172 |
|  | Mobility | 0.26 | 0.613 |
|  | Vigilance | 3.48 | 0.062 |
| Other individual traits | Body weight | 1.99 | 0.159 |
|  | Age category | 0.56 | 0.453 |
| Generic experience |  | 0.50 | 0.479 |
